# Supplementary material for: Bleeding in cardiac patients prescribed antithrombotic drugs: electronic health record phenotyping algorithms, incidence, trends and prognosis
Source: BMC Med. 2019 Nov 20;17:206. doi: 10.1186/s12916-019-1438-y (PMC6864929; doi:10.1186/s12916-019-1438-y)
Supplement: Supplementary file 1 — Additional file 1. Supplementary methods, figures and tables. [file 12916_2019_1438_MOESM1_ESM.docx]

**Supplementary appendix**

| Table S1 | Summary of previous bleeding EHR phenotype algorithms developed using electronic health records |
| --- | --- |
| Table S2 | Bleeding terms from Read and ICD-10 |
| Methods S3 | Development of the CALIBER bleeding EHR phenotype algorithm |
| Figure S4 | Overlap of 39804 bleeding records in CPRD (primary care), HES (secondary care) and ONS (death registry) and the number of inferred bleeding cases in patients without a bleeding record in primary or secondary care (n= 128815 patients) |
| Table S5 | Comparison of results of clinicians review vs. hospitalised bleeding EHR phenotype for identifying bleeding events |
| Table S6 | A summary of 13 false negative cases (algorithm= no bleeding, clinician review = bleeding) in the hospitalised bleeding phenotype algorithm validation sub-study |
| Table S7 | A summary of cases identified to have haemorrhagic transformation |
| Figure S8 | Five year risk of recurrent bleeding stratified by initial bleeding type: any bleeding or bleeding with further markers of severity (bleeding +). A: Risk of any bleeding; B: Risk of fatal or bleeding with further markers of severity |
| Table S9 | Patient baseline characteristics (time of AF, MI, UA or SA diagnosis) stratified by first CALIBER bleeding event type |
| Table S10 | Bleeding definitions used in clinical practice |
| Figure S11 | Short term mortality with and without indicators of bleeding severity |

**Table S1: Summary of previous bleeding EHR phenotypes developed in electronic health records**

| **Author** | **Year** | **Bleeding endpoint(s) evaluated** | **Data source(s)** | **Setting** | **Study Population** | **Coding system**  **(n codes)** | **Supporting EHR data used in case definition** | **Algorithm figure reported** | **Assessment of phenotype accuracy** |
| --- | --- | --- | --- | --- | --- | --- | --- | --- | --- |
| Raiford et al^1^ | 1996 | Upper GI bleeding or perforation | Saskatchewan Hospital Services Plan | Hospital admissions | Patients hospitalised for upper GI bleeding | ICD-9 (30) | No | No | Site specific codes  PPV: 91%  Nonspecific codes  PPV: 68% |
| De Abajo et al^2^ | 1999 | Upper GI bleeding | GPRD  (UK) | Primary care | Patients with a record for acute upper GI bleeding | Read (codes not stated) | No | No | PPV: 95/96 |
| Arnason et al^3^ | 2006 | From 8 anatomical sites:  Any bleeding  Major bleeding | A university hospital, Ottawa | Hospital admissions | Patients with a record for thromboembolism or bleeding | ICD-9 (81) | No  (information from patient charts were used to classify severity) | No | Definite bleeding  PPV: 91%; NPV: 91%  Major bleeding  PPV: 87%; NPV: 92% |
| Wahl et al^4^ | 2010 | Severe upper GI bleeding | HealthCore Integrated Research Database (USA) | Hospital admissions | Patients with a record for upper GI bleeding | ICD-9 (original:75; refined:33) | Procedure codes | No | PPV:  original: 56.5%  refined: 87.8% |
| Cunningham et al^5^ | 2011 | Serious bleeding related to oral anticoagulation from >4 anatomical sites | Tennessee Medicaid program | Hospital admissions | Medicaid enrolees >30 years old | ICD-9 (39) | No  (information from patient charts were used to classify severity) | No | PPV assessed for individual codes ranged from 71.4% to 100% (>5 charts) |
| Crooks et al^6^ | 2012 | Upper GI bleeding | CPRD  HES  ONS  (UK) | Primary care  Hospital admissions  Death registry | Patients with a record for acute upper GI bleeding | Read (46)  ICD-10 (22) | Causes, symptoms, endoscopy, death, transfusion, procedures, alcohol, anaemia, coagulation, collapse, other | Yes | None |
| Valkhoff et al^7^ | 2014 | Upper GI bleeding | IPCI (Netherlands)  HSD (Italy)  ARS (Italy)  Aarhus (Denmark) | Primary care  Hospital admissions | Patients with a record for upper GI bleeding | IPCI (4)  ICD-9 (26)  ICD-10 (16) | No | No | IPCI - PPV: 21%  HSD - PPV: 78%  ARS - PPV: 72%  Aarhus - PPV: 77% |
| Friberg et al^8^ | 2016 | In 4 categories of anatomical site:  Fatal  Non-fatal major  Hospitalised  Minor | Swedish Patient register | Hospital admissions  Hospital outpatients  Death registry | Atrial fibrillation patients | ICD-10 (38) | Anatomical site  (intracranial)  Transfusion  Hospitalisation  Diagnosis position | No | Fatal  PPV: 88.1%; NPV: 99.7%  Non-fatal major  PPV: 90.6%; NPV: 91.5%  Hospitalised  PPV: 65.1%; NPV: 97.5%  Minor  PPV: 84.2%; NPV: 98.9% |
| **CALIBER – present study** | 2017 | In 18 categories of anatomical site:  Fatal  Hospitalised with markers of severity  Hospitalised  Primary care with markers of severity  Primary care  Inferred | CPRD  HES  ONS | Primary care  Hospital admissions  Death registry | Coronary disease and atrial fibrillation patients | Read (201)  ICD-10(96) | Yes: Transfusion, anatomical site; procedures; | Yes | Analysed prognosis following bleeding |

Note: GI= gastrointestinal; PPV= positive predictive value; NPV= negative predictive value

**Table S2: Bleeding Read and ICD-10 codes**

| **Anatomical Site** | **ICD-10 term** | **Read codes** |
| --- | --- | --- |
| Intracranial (Intracerebral) | I61; I610; I611; I612; I613; I614; I615; I616; I618; I619; | G61z.00; G61..00; G614.00; G613.00; G61X100; G61X000; G600.00; G616.00; G617.00; G61X.00; G610.00; G611.00; G612.00; Gyu6200; G618.00; G615.00; Gyu6F00 |
| Intracranial (Subarachnoid) | I60; I600; I601; I602; I603; I604; I605; I606; I607; I608; I609; I62 | G60..00; G604.00; G602.00; G60z.00; G605.00; G603.00; G601.00; G606.00; Gyu6100 |
| Intracranial (Subdural) | I620 | G621.00; G623.00 |
| Intracranial (extradural) | I621; S064 | G620.00; S626.00 |
| Intracranial (unspecified) | I629 | G62z.00; G62..00 |
| Aortic aneurysm or haemopericardium | I230; I312; I711; I713; I715; I718 | G715000; G713.11; G715.00; G530.00; G711.11; G713.00; G360.00; G711.00; G723500; G713000 |
| Upper gastrointestinal | I850; I983; K250; K252; K254; K256; K260; K262; K264; K266; K270; K272; K274; K276; K280; K282; K284; K286; K290; K661; K920 | J110111; J120100; J150000; J110100; J130100; J130300; J120300; J110300; J140100; J680.00; J680.11; J12y100; J68z200; J68z000; J10y000; 1994.11; J121111; 7619100; G850.00; J12yy00; J111111; 4A5..11; 4A23.11; 1994; 1995; J121100; J131100; 4A23.00; J11y100; 4A5..00; J14y100; 4A51.00; J111100; J13y100; 4A5Z.00; J121300; J111300; J12y300; J11yy00; J13y300; G852000 |
| Lower gastrointestinal | K625; K921 | J681.00; J573011; J510900; J68z100; J681.11; SE23111; 19E6.00; 19E6.11; J573012; J573000; SE22300; 196C.00; 196B.00; 479..11; 4762.11; 19E4.12; J573.00; J681.13; J681.12; J573100; 4737.11; 4762; S740100; J573z00 |
| Unspecified gastrointestinal | K922 | J68z.11; J68..00; J68zz00; J68z.00 |
| Genitourinary | N02; N026; N028; N029; N421; N501; N836; N837; N857; N897; N898; N908; N921; N925; N926; N93; N938; N939; N950; R31; R31X | K197.00; K286v00; K5E..00; 1A45.00; K197400; K197300; K19y411; 1584; K197000; K19y400; K59yx00; K56y100; K286w00; K575.00; K197100; K5E1.00; K286100; K5E2.00; K59yy00; K5E0.00; K5Ez.00; K566.00; K55y300; K16y200; K275100; K221100; K286400; K537.00; K167.00; K275200; Kyu9D00 |
| Upper respiratory | R040; R041 | R047.00; R047.11; 1C62.00; 2D25.00; R048.00; 2DE7.00 |
| Lower respiratory | J942; R042 | R063.00; 172..00; R063100; R063000; R063z00 |
| Unspecified respiratory | R048; R049 | - |
| Ocular | H313; H356; H431; H450 | F4K7.00; F4K2800; F404500; F42y500; 2BB8.00; 2BB5.00; F42y.11; F436000; F4Ey000; F436100; F42y400; FyuH400; F436.00; F4H4100; F42y100; F436z00; F424300; F437200; F42y300 |
| Ear | H922 | F503100; F501G00 |
| Renal | - | S760100; K13y800; S760111; K138300; K138100; S761100; C154200 |
| Bleeding disorders | D683; D69; D698; D699 | D31..00; D31z.00; D31X.00; D31yz00; D31y.00; Dyu3300 |
| Unspecified | R233; R58; R58X; T81 | SE...11; SE33011; SE4z.11; SE45.11; SK02.12; SK02.00; SE4z.12; S750100; 2F65.00; SK02.11; S751100; Ryu7300 |

**Methods S3: Development of the CALIBER bleeding EHR phenotype algorithm**

*1) Reviewing code lists for bleeding and related procedures*

We reviewed bleeding terms in Read and ICD-10 to define bleeding in primary and hospital care and to determine which might be relevant to antithrombotic use. We identified 96 ICD-10 codes and 201 Read codes (**Additional file 1: Table S2**) for bleeding events which we categorised into 18 anatomical sites [Intracranial (intracerebral, subarachnoid, extradural, subdural, unspecified); Gastrointestinal (upper, lower, unspecified); Respiratory (upper, lower, unspecified); Ruptured aortic aneurysm or haemopericardium; Genitourinary; Bleeding disorder; Ocular; Ear; Renal; Unspecified]. We used the field related to bleeding complications in MINAP, which consists of 6 categories pertaining to location and haemoglobin drop. Furthermore we reviewed primary care Read codes and hospital care OPCS codes for procedures including transfusion, haematoma evacuation or aspiration, surgical arrest for bleeding and endoscopy. All codes used to derive the bleeding phenotype are available at the CALIBER portal **[**<https://www.caliberresearch.org/portal>].

*2) Preliminary analysis*

We performed a preliminary analysis of the bleeding records in our study population to determine which data are viable for use in the final algorithm. First we examined the characteristics of the population at entry by whether they had a bleeding or transfusion code in primary or hospital care, or bleeding listed as a cause of death in follow up. We examined the characteristics of bleeding records stratified by anatomical bleeding site. For hospitalised bleeding we calculated the length of hospitalisation and the proportion of records with primary diagnosis position. We investigated the presence of procedures records (transfusion, bleeding surgical arrest, haematoma evacuation and endoscopy) at different time intervals (on the same day, within 7 days and within 30 days) of bleeding records in primary and hospital care. Haemoglobin drop related to a bleeding event was calculated as the peak haemoglobin value within 365 to 7 days prior to bleeding minus the lowest haemoglobin value recorded within 7 days of bleeding for patients with a minimum of 2 haemoglobin values recorded in primary care.

*3) Markers of bleeding severity*

To assess the suitability of markers for bleeding severity we examined short term (30 and 90 days) all-cause and bleeding specific mortality following bleeding with and without the indicators. Guided by current clinical bleeding definitions **(Additional file 1: Table S10)** and availability of data within CALIBER the following severity indicators were considered, 1) anatomical site, 2) presence of a transfusion record within 30 days, 3) presence of surgical interventions within 30 days, 4) haemoglobin drop and 5) bleeding from more than one site on a single date. Furthermore for hospitalised bleeding records we considered primary versus secondary diagnosis position and length of hospitalisation.

The Kaplan-Meier analysis of severity markers and short term outcomes is shown in **Additional file 1: Figure S11**. These were used to inform the definition of severity in the algorithm. For example, transfusion records in primary care were uncommon and only showed modest signs of associated increased short term mortality and therefore only transfusions recorded in hospital care are used to define severity in the algorithm**.**

*4) Inferring bleeding cases*

We attempted to capture potential bleeding cases in patients with no bleeding code in primary or hospital care through the following pathways:

- Surgical procedures (surgical arrest, haematoma evacuation) recorded in primary and hospital care
- Transfusion with
  - Iron deficiency anaemia record in primary care or hospital within 30 days
  - Low haemoglobin (<10g/dL) and endoscopy within 30 days and no cancer, liver disease, renal disease diagnosis 1 year prior
- Low haemoglobin (<10g/dL) with
  - Iron deficiency anaemia record in primary care or hospital care and endoscopy within 30 days and no cancer, liver disease, renal disease diagnosis 1 year prior

*5) Developing the phenotype*

Based on a combination of exploratory analyses of bleeding codes and severity markers and consensus amongst the study team we iteratively developed an algorithm to define bleeding in linked EHR. We grouped bleeding as major and minor both within primary and hospital care, or inferred.

We assessed how well bleeding events are captured amongst the data sources used, allowing up to 30 days between bleeding events in different sources to be considered the same event, using a Venn diagram.

**Figure S4: Overlap of 39,804 bleeding recorded in CPRD (primary care), HES (hospital admissions) and ONS (death registry) and the number of inferred bleeding cases in patients without a bleeding record in primary or hospital care (n= 128,815 patients)**

67

4689

445

60

**HES**

**CPRD**

**ONS**

Bleeding recorded

Bleeding Inferred

**Note: Numbers of inferred (possible) bleeding events according to source of information: 477 surgical arrest or haematoma evacuation procedures in 451 patients; in 514 patients 689 cases of a transfusion code in OPCS accompanied by an iron deficiency anaemia diagnosis in HES or CPRD within 30 days; in 62 patients 77 cases of a transfusion code in OPCS accompanied by a haemoglobin value of <10g/dL in CPRD within 30 days, an endoscopic examination within 30 days and no history of cancer, liver or renal disease in the year prior to transfusion; and in 182 patients 249 cases of haemoglobin <10g/dL in CPRD, an endoscopic examination within 30 days and no history of cancer, liver or renal disease in the year prior to the haemoglobin record. That is, overall 1,492 potential bleeding events identified in 1144/101,566 (1.1%) patients with no bleeding record in HES or CPRD.**

**Table S5: Comparison of results of clinicians review vs. hospitalised bleeding EHR phenotype for identifying bleeding events**

| **Bleeding phenotype algorithm** | **Clinician review** | |  |
| --- | --- | --- | --- |
|  | Bleeding | No bleeding | Total |
| Bleeding | 15 | 2 | 17 |
| No bleeding | 6 | 260 | 266 |
| Total | 21 | 262 | 283 |

PPV: 0.88, 95% CI: 0.64, 0.99

NPV: 0.98, 95% CI: 0.95, 0.99

Sensitivity: 0.71, 95% CI: 0.48, 0.89

Specificity: 0.99, 95%CI: 0.97, 1.00

**Note:** **The hospitalised bleeding part of the bleeding phenotype algorithm (ICD-10 diagnosis codes and OPCS-4 procedure codes) was validated in a cohort of 283 stroke related hospitalisations. Two clinicians, blinded to the codes recorded, reviewed the record corpus for each hospitalistion and adjudicated whether bleeding occurred. Bleeding assignments from the clinicians review was compared with those from the bleeding algorithm and positive predictive value (PPV), negative predictive value (NPV), sensitivity and specificity using the case review data as the gold standard.**

**Table S6: A summary of 13 false negative cases (algorithm= no bleeding, clinician review = bleeding) in the hospitalised bleeding phenotype algorithm validation sub-study**

| **Case** | **ICD-10 and OPCS-4 codes recorded during hospitalisation** | **Clinician review of discharge notes** |
| --- | --- | --- |
| 1 | T828 - Other specified complications of cardiac and vascular prosthetic devices, implants and grafts  Y841 - Kidney dialysis  I120 - Hypertensive renal disease with renal failure  E102 - Type 1 diabetes mellitus  N083 - Glomerular disorders in diabetes mellitus  I951 - Orthostatic hypotension  I501 - Left ventricular failure  I258 - Other forms of chronic ischaemic heart disease  D649 - Anaemia, unspecified  E785 - Hyperlipidaemia, unspecified  M1096 - Gout, unspecified  Z602 - Living alone  Z955 - Presence of coronary angioplasty implant and graft  Z880 - Personal history of allergy to penicillin  Z864 - Personal history of psychoactive substance abuse | GI Bleed - Duodenal Ulcer |
| 2 | N390 - Urinary tract infection, site not specified  E854 - Organ-limited amyloidosis  I680 - Cerebral amyloid angiopathy  E119 - Type 2 diabetes mellitus | Haematemesis. |
| 3 | N132 - Hydronephrosis with renal and ureteral calculous obstruction  N134 – Hydroureter  N390 - Urinary tract infection, site not specified  J459 - Asthma, unspecified  M273 - Ureteroscopic extraction of calculus of ureter  Y149 - Unspecified placement of stent in organ NOC  Z943 - Left sided operation  U212 - Computed tomography NEC  Y981 - Radiology of one body area (or < 20 minutes)  Z411 - Kidney  Z413 - Ureter NEC  Z421 - Bladder NEC | Haematuria |
| 4 | I639 - Cerebral infarction, unspecified  Q273 - Peripheral arteriovenous malformation  U114 - Computed tomography scan of cerebral vessels  Y973 - Radiology with post contrast  Y981 - Radiology of one body area (or < 20 minutes) | Major PR bleeding. |
| 5 | I634 - Cerebral infarction due to embolism of cerebral arteries  Y445 - Thrombolytic drugs  I672 - Cerebral atherosclerosis  I440 - Atrioventricular block, first degree  M139 - Arthritis, unspecified  L343 - Open embolectomy of cerebral artery  Y534 - Approach to organ under fluoroscopic control  U051 - Computed tomography of head  Y981 - Radiology of one body area (or < 20 minutes)  X833 - Fibrinolytic drugs Band 1 | 2 episodes of significant haemoptysis |
| 6 | I639 - Cerebral infarction, unspecified  I489 - Atrial fibrillation and atrial flutter, unspecified  I10X - Essential (primary) hypertension  J449 - Chronic obstructive pulmonary disease, unspecified  K219 - Gastro-oesophageal reflux disease without oesophagitisM199 - Arthrosis, unspecified  M419 - Scoliosis, unspecified  Z950 - Presence of electronic cardiac devices  Z921 - Personal history of long-term (current) use of anticoagulants  Z880 - Personal history of allergy to penicillin  Z881 - Personal history of allergy to other antibiotic agents | Superficial bruising + haematoma on UL + shins. |

**Table S7: A summary of cases identified to have haemorrhagic transformation**

| **Case** | **ICD-10 and OPCS-4 codes recorded during hospitalisation** | **Clinician review of discharge notes** |
| --- | --- | --- |
| 1 | R208 - Other and unspecified disturbances of skin sensation  R51X - Headache  Z922 - Personal history of long-term (current) use of other medicaments | CT head report-“with previous haemorrhage and new micro haemorrhagic transformation” |
| 2 | I633 - Cerebral infarction due to thrombosis of cerebral arteries  I630 - Cerebral infarction due to thrombosis of precerebral arteries  E780 - Pure hypercholesterolaemia  Q600 - Renal agenesis, unilateral | Ct head report:”haemorrhagic transformation of stroke”. |
| 3 | I639 - Cerebral infarction, unspecified  G819 - Hemiplegia, unspecified  E039 - Hypothyroidism, unspecified  X833 - Fibrinolytic drugs Band 1  U051 - Computed tomography of head  Y981 - Radiology of one body area (or < 20 minutes) | CT head report:”haemorrhagic transformation within the Left Basal ganglia”. |
| 4 | I633 - Cerebral infarction due to thrombosis of cerebral arteries  R798 - Other specified abnormal findings of blood chemistry  F171 - Mental and behavioural disorders due to use of tobacco  Z722 - Drug use  U051 - Computed tomography of head  Y981 - Radiology of one body area (or < 20 minutes)  U212 - Computed tomography NEC  Y973 - Radiology with post contrast  Y982 - Radiology of two body areas  Z921 - Head NEC  Z923 - Neck NEC  U201 - Transthoracic echocardiography | Ct head report:”haemorrhagic transformation of stroke”. |
| 5 | I639 - Cerebral infarction, unspecified  G819 - Hemiplegia, unspecified  U051 - Computed tomography of head  Y981 - Radiology of one body area (or < 20 minutes) | CT head report: “RMCA infarct haemorrhagic transformation”. |
| 6 | NONE RECORDED | CT head report: “microhaemorrhages and haemorrhagic transformation”. |
| 7 | NONE RECORDED | CT head report: “haemorrhagic transformation”. |

**Figure S8: Five year risk of recurrent bleeding stratified by initial bleeding type: any bleeding or bleeding with further markers of severity (bleeding +). A: Risk of any bleeding; B: Risk of fatal or bleeding with further markers of severity**


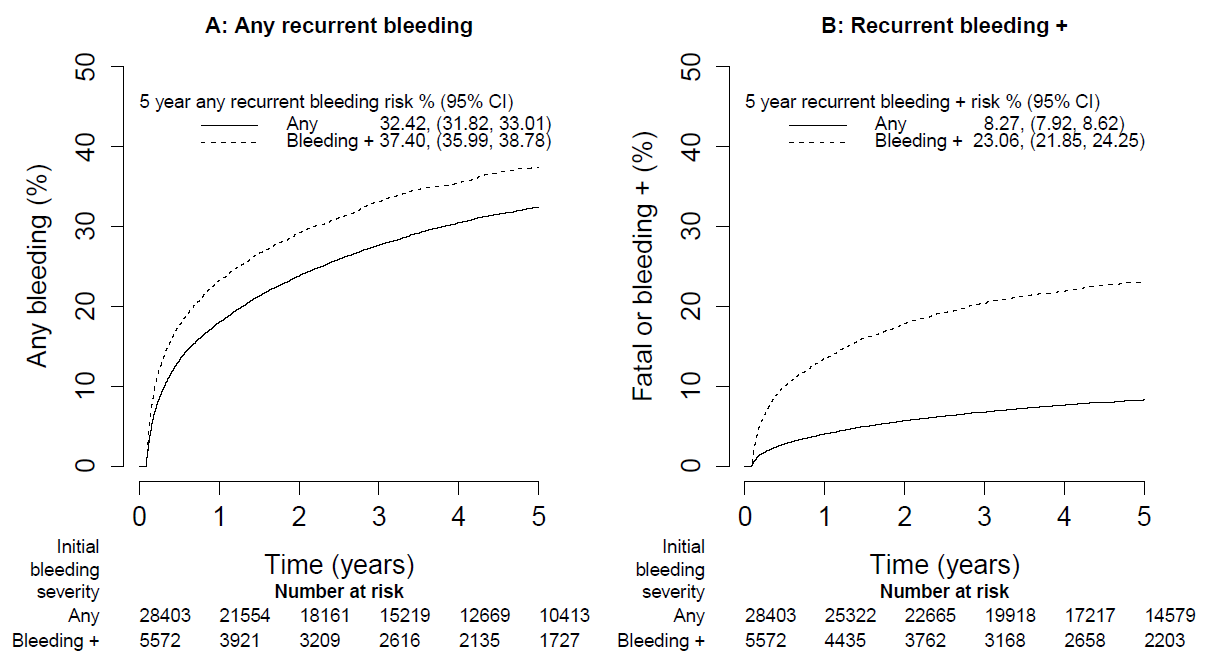


**Note:** ‘Any bleeding’ includes hospitalised, hospitalised +, primary care, primary care + and inferred bleeding. ‘Bleeding +’ includes hospitalised + or primary care + bleeding

**Table S9: Patient baseline characteristics (time of Atrial fibrillation, Myocardial infarction, Unstable angina or Stable angina diagnosis) stratified by first CALIBER bleeding event type**

|  |  | **I** | **II** | | | **III** |  |
| --- | --- | --- | --- | --- | --- | --- | --- |
|  | **Fatal (n=1575)** | **Hospitalised +MS**  **(n=2654)** | **Primary care +MS (n=1645)** | **Hospitalised (n=8766)** | **Inferred**  **(n=1144)** | **Primary care (n=12619)** | **No bleeding (n=100412)** |
| **Demographics and behaviours**  **at cohort entry** |  |  |  |  |  |  |  |
| Age (years), mean (SD) | 77.4 (9.76) | 75.6 (10.98) | 73.6 (10.81) | 71.5 (11.86) | 74.1 (11.48) | 70.1 (11.88) | 71.4 (13.31) |
| Women, n (%) | 690 (43.8) | 1224 (46.1) | 680 (41.3) | 3977 (45.4) | 549 (48.0) | 5124 (40.6) | 46762 (46.6) |
| Highest quartile of deprivation (most deprived) | 341 (21.7) | 526 (19.9) | 292 (17.8) | 1922 (22.0) | 239 (20.9) | 2322 (18.4) | 20037 (20.0) |
| *% missing* | 0.2 | 0.3 | 0.1 | 0.3 | 0.3 | 0.2 | 0.3 |
| Smoking status, n (%) |  |  |  |  |  |  |  |
| Non-Smoker | 631 (52.6) | 1149 (56.9) | 718 (57.3) | 3751 (54) | 484 (54.4) | 5527 (54.9) | 41145 (51.1) |
| Current smoker | 160 (13.3) | 183 (9.1) | 97 (7.7) | 785 (11.3) | 104 (11.7) | 1006 (10.0) | 10920 (13.6) |
| Ex-smoker | 409 (34.1) | 688 (34.1) | 439 (35.0) | 2413 (34.7) | 301 (33.9) | 3535 (35.1) | 28478 (35.4) |
| *% missing* | 23.8 | 23.9 | 23.8 | 20.7 | 22.3 | 20.2 | 19.8 |
| History of alcohol abuse, n (%) | 125 (7.9) | 265 (10.0) | 159 (9.7) | 868 (9.9) | 115 (10.1) | 1203 (9.5) | 9689 (9.6) |
| **Medical history**  ***(Any record ever prior to entry)*** |  |  |  |  |  |  |  |
| Myocardial infarction, n (%) | 290 (18.4) | 486 (18.3) | 296 (18.0) | 1508 (17.2) | 190 (16.6) | 2098 (16.6) | 21778 (21.7) |
| Atrial fibrillation, n (%) | 411 (26.1) | 677 (25.5) | 303 (18.4) | 1685 (19.2) | 295 (25.8) | 1870 (14.8) | 21820 (21.7) |
| Stable angina, n (%) | 820 (52.1) | 1372 (51.7) | 959 (58.3) | 5115 (58.4) | 613 (53.6) | 7930 (62.8) | 52960 (52.7) |
| Unstable angina, n (%) | 129 (8.2) | 251 (9.5) | 157 (9.5) | 900 (10.3) | 114 (10.0) | 1271 (10.1) | 9073 (9.0) |
| Diabetes, n (%) |  |  |  |  |  |  |  |
| Type 1 | 10 (0.6) | 31 (1.2) | 21 (1.3) | 95 (1.1) | 11 (1.0) | 112 (0.9) | 858 (0.9) |
| Type 2 | 164 (10.4) | 367 (13.8) | 208 (12.6) | 1124 (12.8) | 180 (15.7) | 1406 (11.1) | 12118 (12.1) |
| Unspecified type | 39 (2.5) | 58 (2.2) | 30 (1.8) | 202 (2.3) | 28 (2.4) | 198 (1.6) | 1951 (1.9) |
| Stroke (ischaemic or unspecified), n (%) | 133 (8.4) | 251 (9.5) | 97 (5.9) | 527 (6.0) | 80 (7.0) | 621 (4.9) | 5915 (5.9) |
| Peripheral arterial disease, n (%) | 205 (13.0) | 312 (11.8) | 174 (10.6) | 850 (9.7) | 136 (11.9) | 1068 (8.5) | 8742 (8.7) |
| Renal disease, n (%) | 143 (9.1) | 235 (8.9) | 144 (8.8) | 659 (7.5) | 91 (8.0) | 658 (5.2) | 7416 (7.4) |
| Cancer, n (%) | 233 (14.8) | 477 (18.0) | 366 (22.2) | 1354 (15.4) | 194 (17.0) | 1777 (14.1) | 14040 (14.0) |
| Peptic ulcer, n (%) | 142 (9.0) | 244 (9.2) | 173 (10.5) | 792 (9.0) | 78 (6.8) | 1023 (8.1) | 6902 (6.9) |
| Bleeding diatheses or coagulation disorders, n (%) | 25 (1.6) | 38 (1.4) | 31 (1.9) | 103 (1.2) | 15 (1.3) | 109 (0.9) | 777 (0.8) |
| Chronic anaemia, n (%) | 260 (16.5) | 491 (18.5) | 453 (27.5) | 1305 (14.9) | 372 (32.5) | 1303 (10.3) | 12929 (12.9) |
| **Biomarkers**  ***(Nearest record to entry within 1 year prior)*** |  |  |  |  |  |  |  |
| Systolic blood pressure (mmHg), mean (SD) | 143 (21.5) | 144 (21.4) | 144 (22.2) | 142 (21.1) | 142 (21.6) | 143 (20.8) | 141 (20.9) |
| *% missing* | 26.0 | 27.9 | 24.9 | 26.2 | 26.1 | 23.7 | 26.1 |
| Haemoglobin (g/dL), mean(SD) | 13.1 (1.90) | 12.9 (1.96) | 12.5 (2.19) | 13.4 (1.81) | 11.7 (2.74) | 13.7 (1.60) | 13.4 (1.80) |
| *% missing* | 62.6 | 61.6 | 59.5 | 61.7 | 57.5 | 62.2 | 59.6 |
| Creatinine (mol/l), median (IQR) | 105 (87, 129) | 100 (84, 122) | 101 (86, 123) | 96 (82, 114) | 101 (85, 123) | 95 (82, 111) | 94 (81, 112) |
| Min, Max | 23.00, 733 | 4.00, 1290 | 8.60, 906 | 1.00, 1036 | 45.00, 739 | 2.60, 919 | 0.11, 1625 |
| *% missing* | 53.5 | 55.1 | 53.1 | 54.1 | 50.6 | 54.2 | 50.9 |
| Body mass index, mean (SD) | 27.0 (5.57) | 27.4 (5.49) | 28.2 (6.07) | 28.3 (5.66) | 27.3 (4.90) | 28.5 (5.44) | 28.1 (5.76) |
| Underweight | 15 (3.1) | 21 (2.6) | 11 (1.9) | 51 (1.7) | 8 (2.0) | 60 (1.4) | 893 (2.6) |
| Normal | 161 (33.8) | 271 (33.2) | 151 (26.5) | 792 (27.1) | 122 (31.2) | 1084 (24.6) | 9416 (27.9) |
| Overweight | 192 (40.3) | 308 (37.7) | 237 (41.7) | 1179 (40.4) | 166 (42.5) | 1824 (41.4) | 12775 (37.8) |
| Obese | 109 (22.9) | 216 (26.5) | 170 (29.9) | 897 (30.7) | 95 (24.3) | 1433 (32.6) | 10675 (31.6) |
| *% missing* | 69.7 | 69.3 | 65.4 | 66.7 | 65.8 | 65.1 | 66.4 |
| **Prescribed antithrombotic therapies and duration**  **between cardiac disease diagnosis and 1^st^ bleeding event (median, IQR)** |  |  |  |  |  |  |  |
| No antithrombotic therapy, n (%) | 436 (27.7) | 591 (22.3) | 402 (24.4) | 1554 (17.7) | 453 (39.6) | 2012 (15.9) | 22983 (22.9) |
| Aspirin monotherapy, n (%) | 852 (54.1) | 1547 (58.3) | 961 (58.4) | 5473 (62.4) | 514 (44.9) | 8344 (66.1) | 64564 (64.3) |
| Duration (days) | 619 (180, 1317) | 538 (179, 1247) | 459 (163, 1098) | 524 (170, 1184) | 427 (127, 1018) | 557 (185, 1228) | 820 (288, 1768) |
| Clopidogrel monotherapy, n (%) | 128 (8.1) | 209 (7.9) | 139 (8.4) | 876 (10.0) | 76 (6.6) | 1264 (10.0) | 11031 (11.0) |
| Duration (days) | 131 (40.5, 550) | 128 (38.0, 422) | 174 (46.0, 558) | 117 (35.0, 448) | 110 (58.8, 447) | 121 (40.0, 440) | 146 (43.0, 567) |
| Dual antiplatelet therapy, n (%) | 166 (10.5) | 339 (12.8) | 188 (11.4) | 1330 (15.2) | 115 (10.1) | 1843 (14.6) | 18242 (18.2) |
| Duration (days) | 216 (90.0, 476) | 174 (77.5, 402) | 164 (80.2, 398) | 197 (90.0, 423) | 148 (77.0, 376) | 180 (90.0, 402) | 322 (118.0, 486) |
| VKA monotherapy, n (%) | 261 (16.6) | 479 (18.0) | 239 (14.5) | 1439 (16.4) | 133 (11.6) | 1892 (15.0) | 11512 (11.5) |
| Duration (days) | 274 (99, 892) | 411 (108, 916) | 280 (99, 656) | 313 (105, 806) | 267 (90, 782) | 292 (97, 775) | 387 (132, 1036) |
| VKA + 1 antiplatelet, n (%) | 139 (8.8) | 205 (7.7) | 121 (7.4) | 740 (8.4) | 59 (5.2) | 1038 (8.2) | 6656 (6.6) |
| Duration (days) | 76 (41.0, 171) | 90 (49.0, 218) | 90 (57.0, 180) | 90 (48.0, 232) | 104 (61.5, 434) | 90 (52.0, 217) | 90 (52.0, 200) |
| VKA + 2 antiplatelets, n (%) | 20 (1.3) | 26 (1) | 15 (0.9) | 77 (0.9) | 3 (0.3) | 104 (0.8) | 874 (0.9) |
| Duration (days) | 70.0 (34.8, 91.0) | 41.5 (19.2, 68.8) | 44.0 (16.0, 79.5) | 59.0 (38.0, 90.0) | 69.0 (44.5, 79.5) | 57.5 (35.0, 90.0) | 64.0 (40.0, 90.0) |

Note: MS= markers of severity; SD= standard deviation; IQR= interquartile range; VKA= vitamin K antagonist

**Table S10: Bleeding definitions used in clinical trials and observational studies and factors used to classify severity**

| Factor | CALIBER – present study | Stable post-MI risk prediction[^9^](#_ENREF_9) | Bleeding Academic Research Consortium (BARC)[^10^](#_ENREF_10) | International Society on Thrombosis and Haemostasis (ISTH)[^11^](#_ENREF_11) | Thrombosis In Myocardial Infarction (TIMI)[^12^](#_ENREF_12) |
| --- | --- | --- | --- | --- | --- |
| Fatal | ● | ● | ● | ● | ● |
| Anatomic location | ●Intracranial; Ruptured aortic aneurysm; Haemopericardium | ●Intracranial | ●Intracranial; Intraocular | ● Intracranial; Intraspinal; Intraocular; Retroperitoneal; Intraarticular; Pericardial; Intramuscular w. compartment syndrome | ● Intracranial |
| Haemoglobin drop | ○ | ○ | ● 3 - <5g/dL; ≥5g/dL | ● ≥2 g/dL | ● 3 - <5g/dL; ≥5g/dL |
| Hospitalisation | ● | ● | ● | ● | ○ |
| Mode of hospital admission | ● | ○ | ○ | ○ | ○ |
| Length of hospitalisation | ● >14 days | ● > 14 days | ○ | ○ | ○ |
| Blood transfusion | ● | ● | ● | ● | ● |
| Number of units transfused | ○ | ○ | ● | ● | ○ |
| Medical/surgical consultation | ● | ○ | ● | ● | ● |
| Medical or surgical intervention | ● | ○ | ● | ● | ● |
| Multiple bleeding codes | ● | ○ | ○ | ○ | ○ |
| Haemodynamic compromise | ○ | ○ | ○ | ● | ○ |
| Change in antithrombotic therapy | ○ | ○ | ○ | ● | ● |

**Figure S11: Short term mortality with and without indicators of bleeding severity**

| Bleeding location | 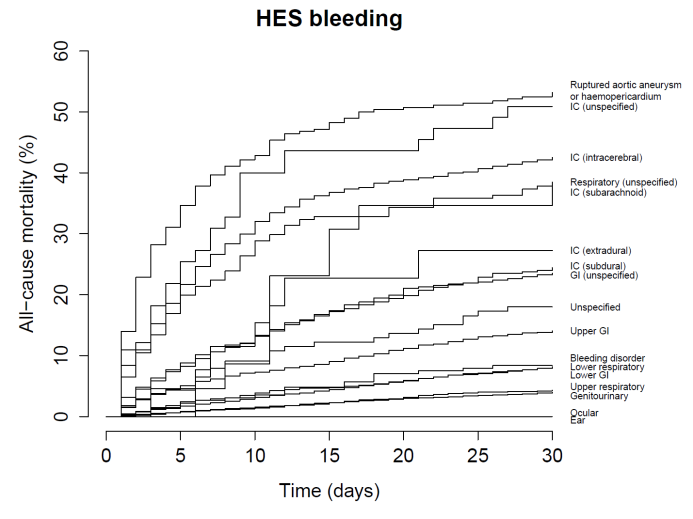 | 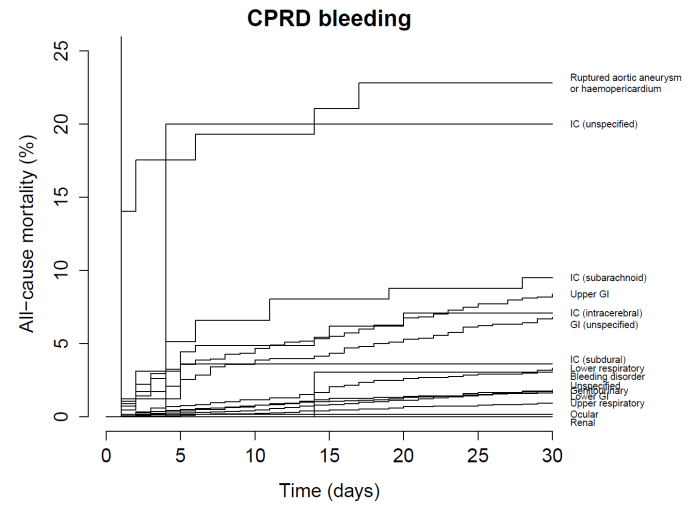 |
| --- | --- | --- |
| Hospitalisation diagnosis position | 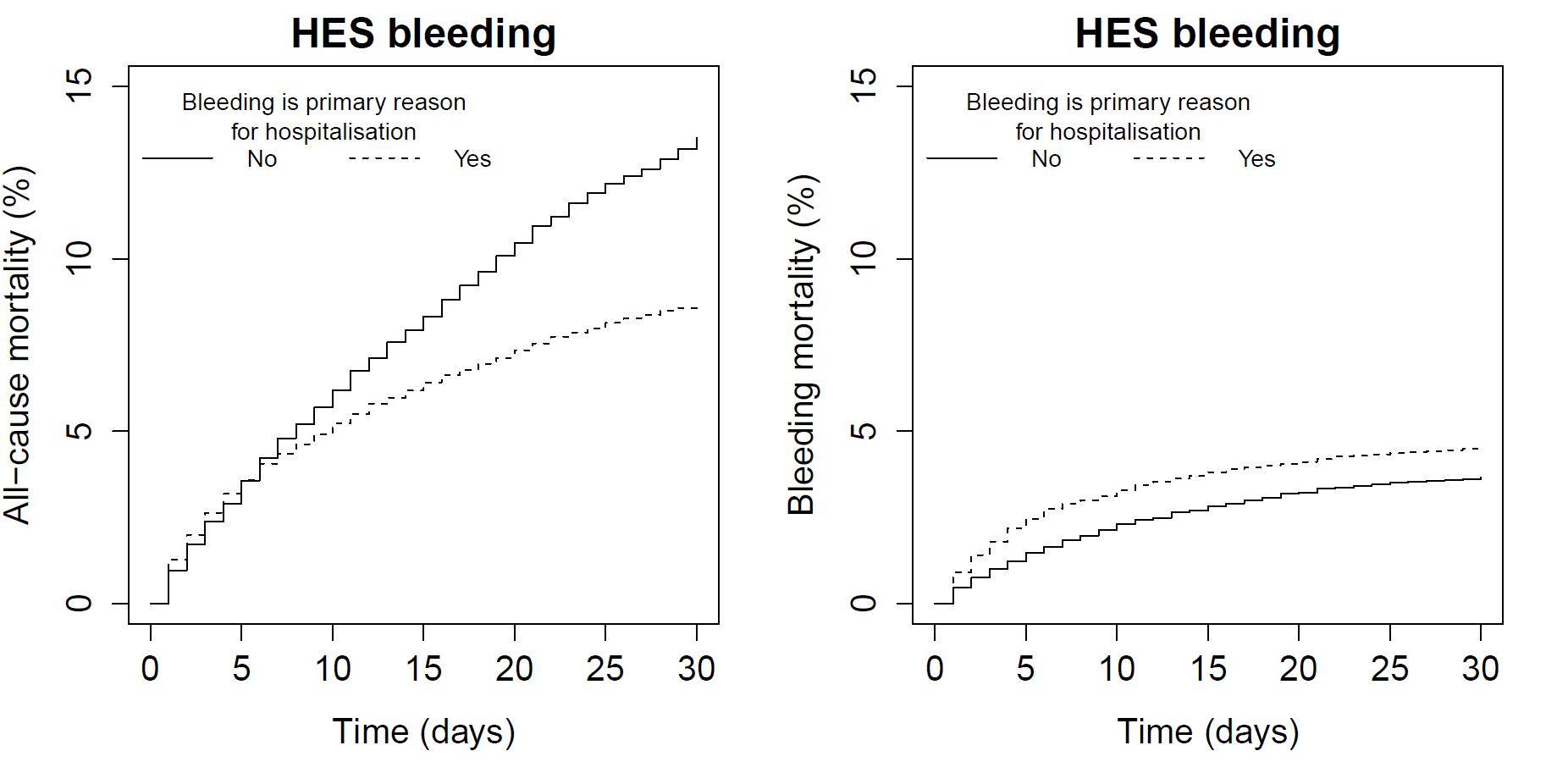 | 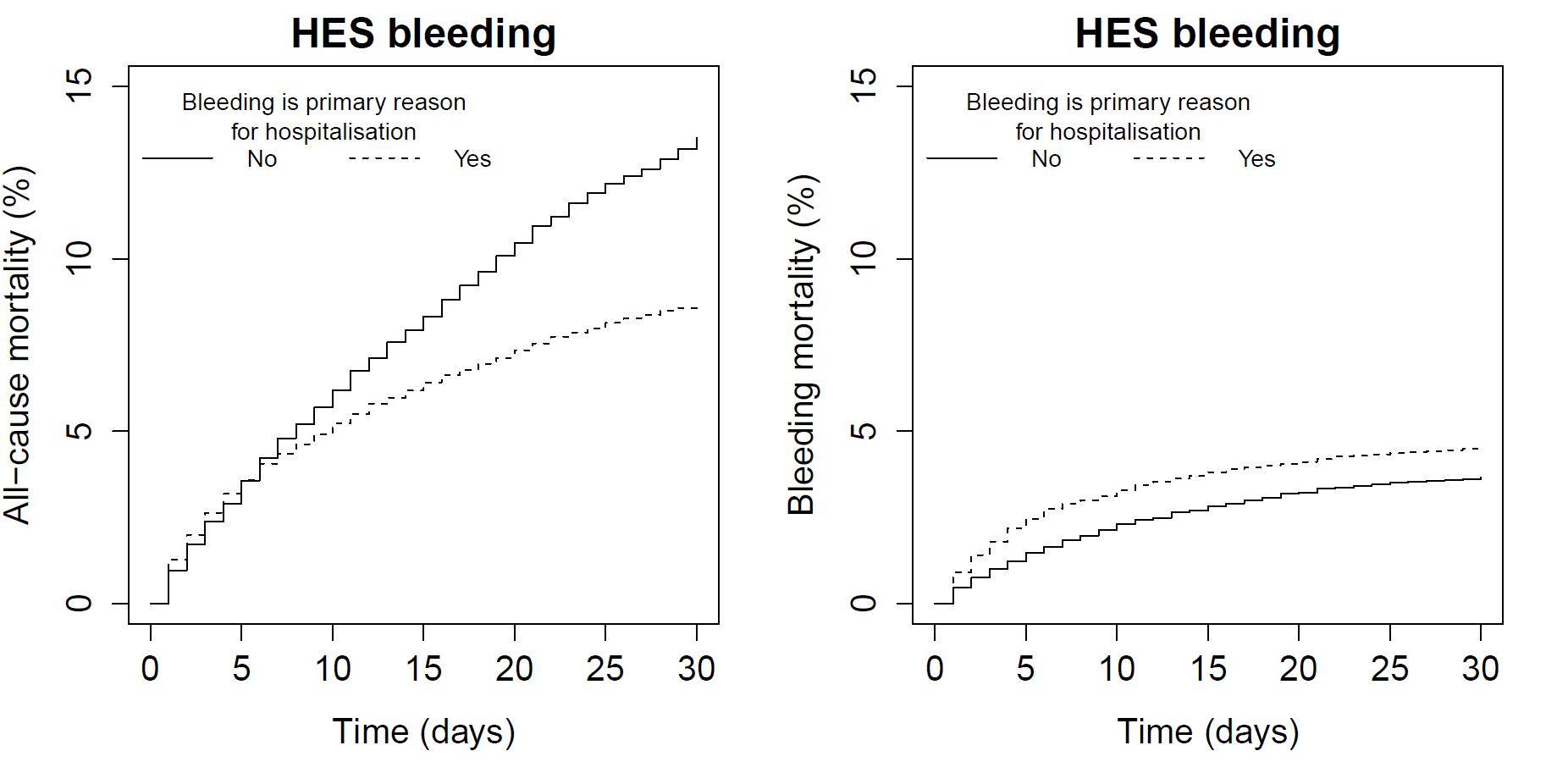 |
| Transfusion | 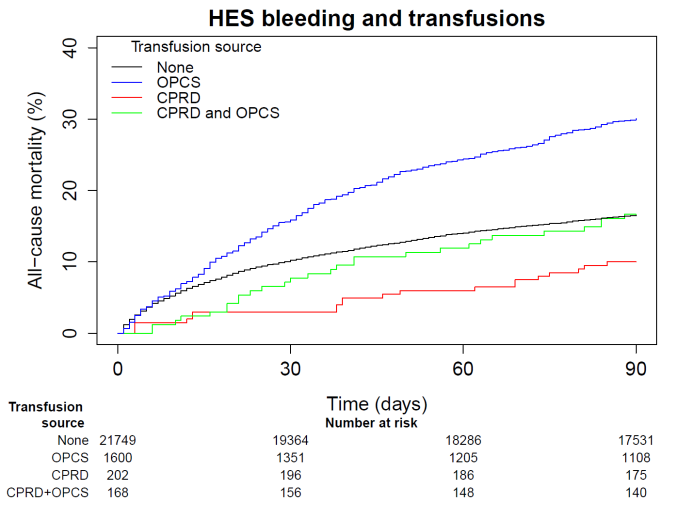 | 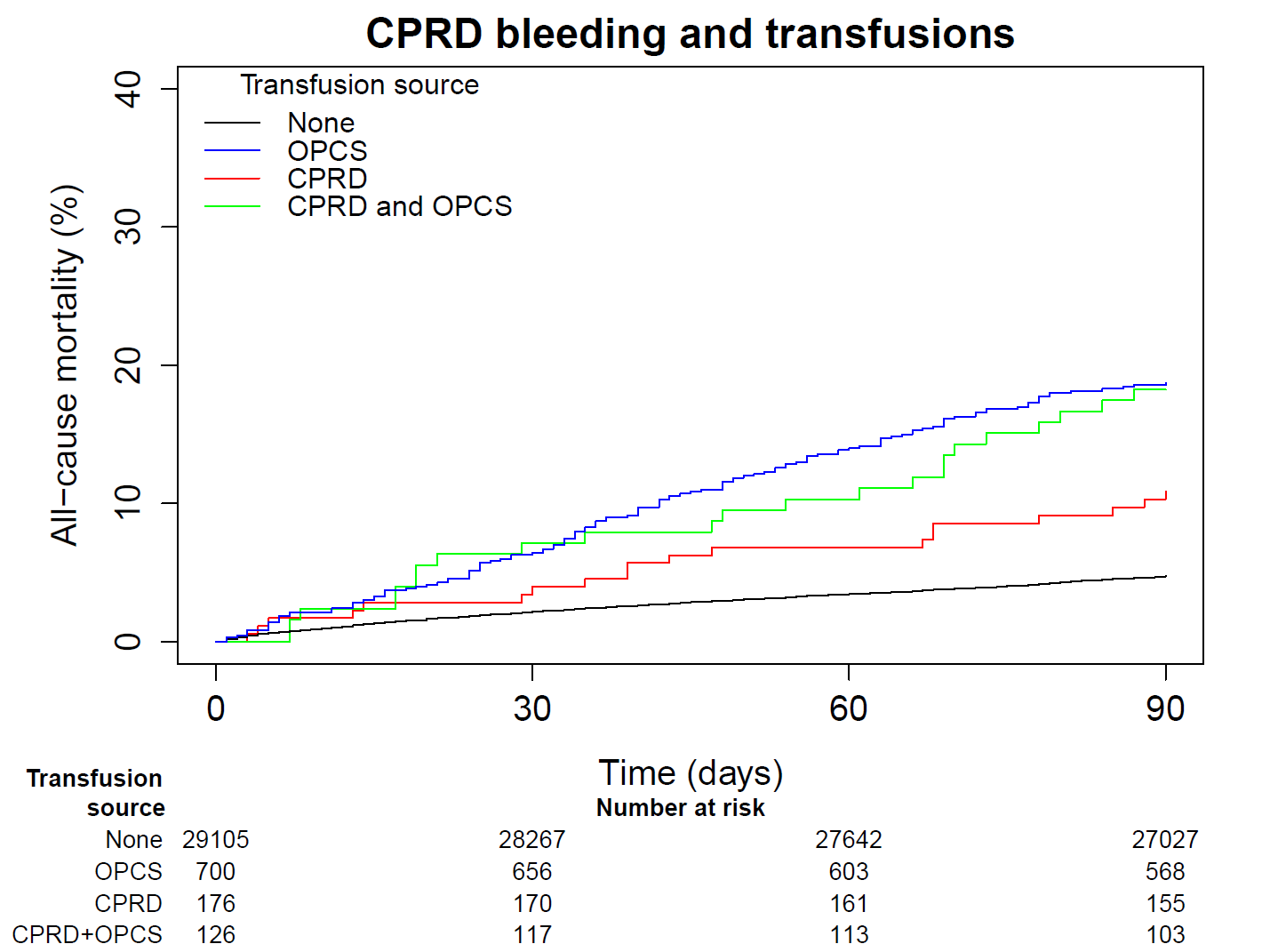 |
| Haemoglobin drop | 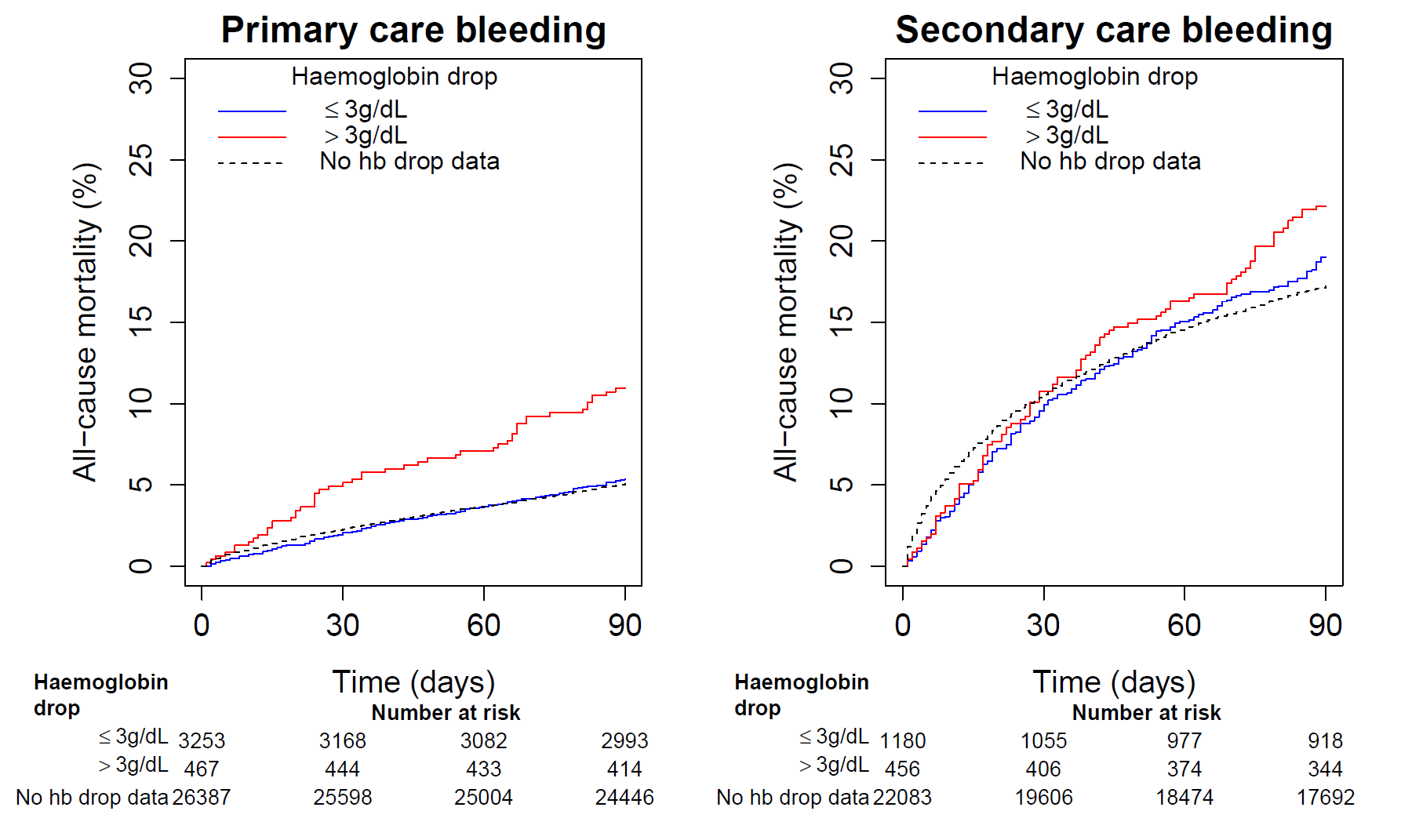 | 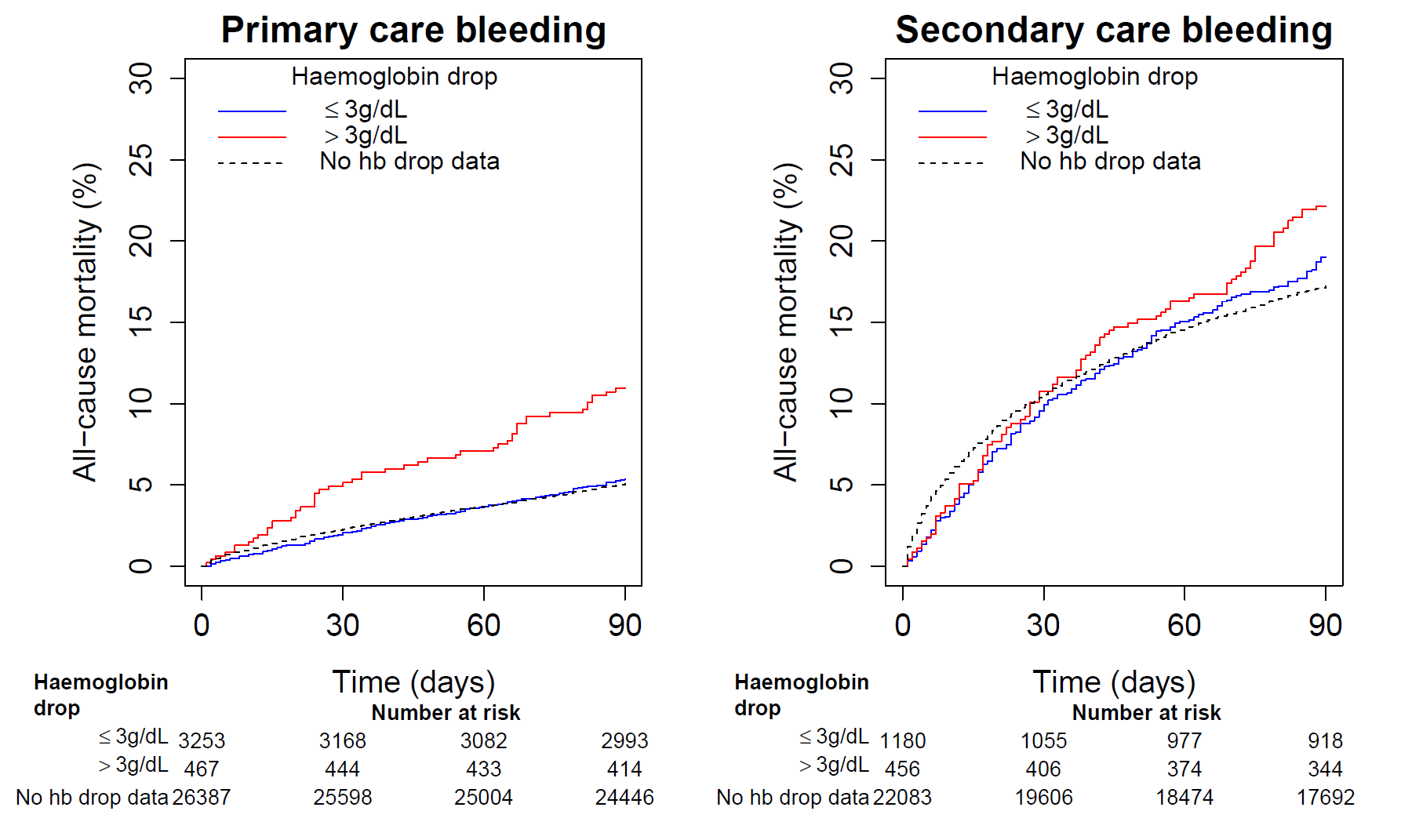 |
| Number of bleeding codes | 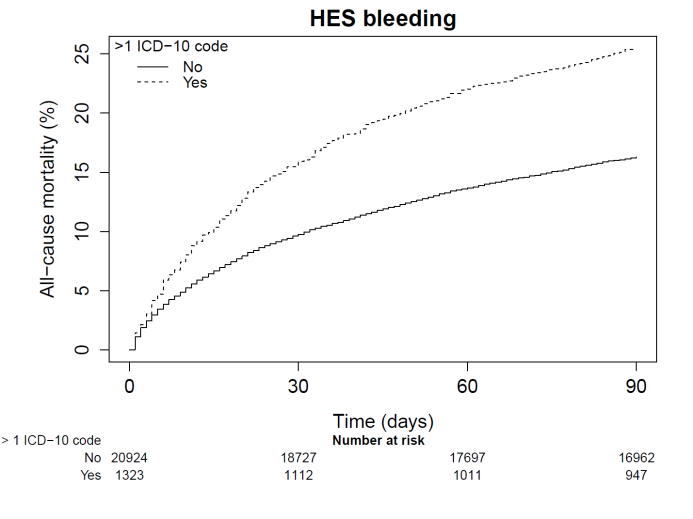 | 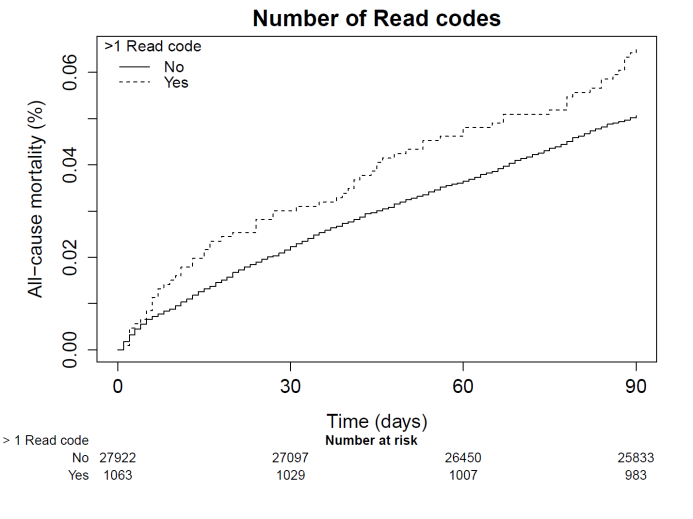 |
| Endoscopy | 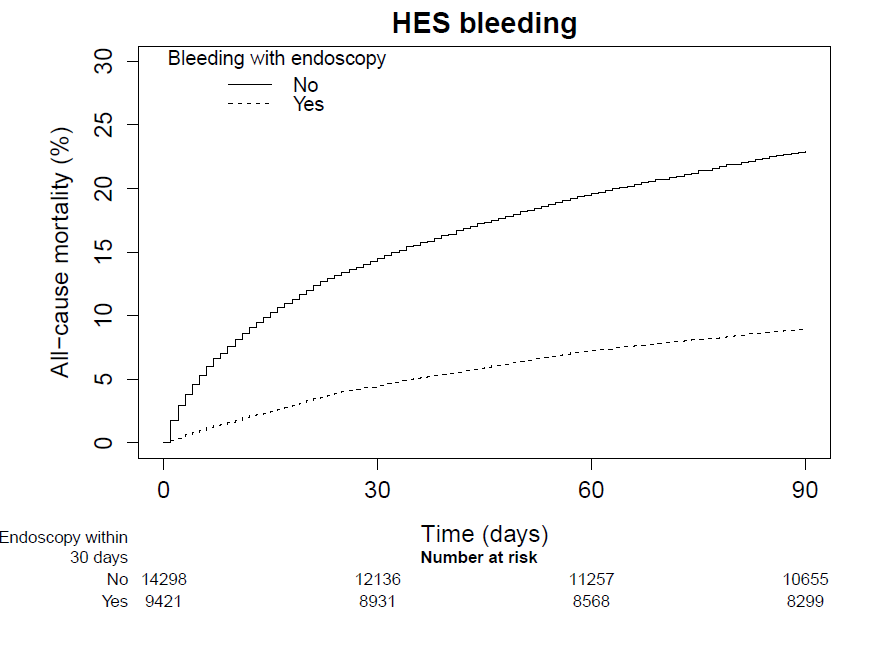 |  |
| Bleeding intervention procedures | 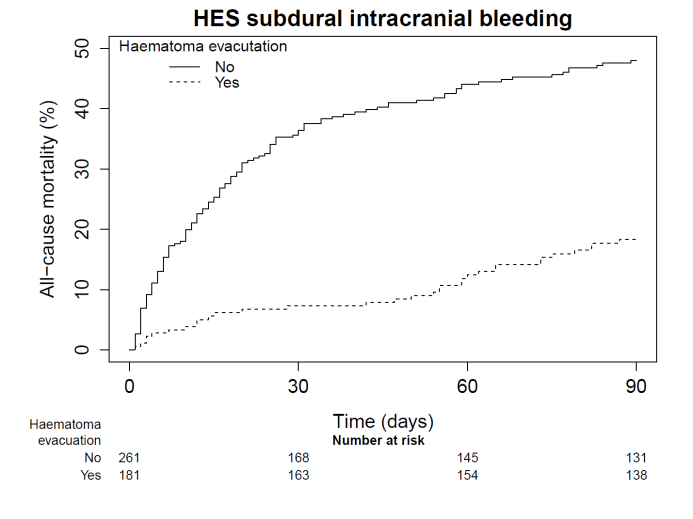 | 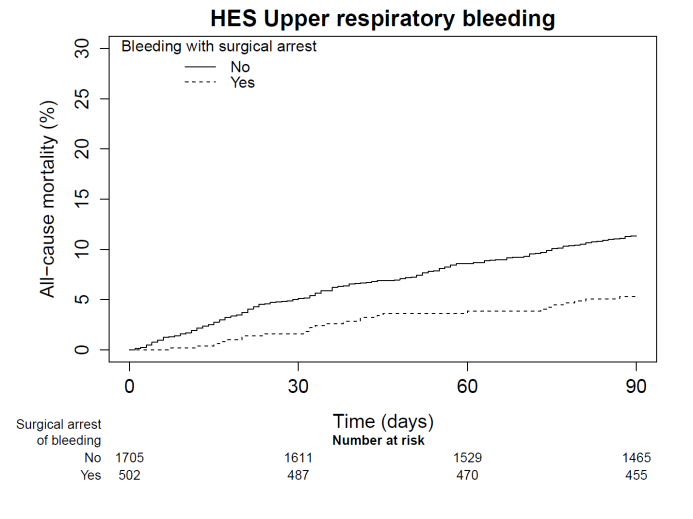 |

**Supplementary appendix references**

1. Raiford DS, Perez Gutthann S, Garcia Rodriguez LA. Positive predictive value of ICD-9 codes in the identification of cases of complicated peptic ulcer disease in the Saskatchewan hospital automated database. *Epidemiology (Cambridge, Mass).* 1996;7(1):101-104.

2. de Abajo FJ, Rodriguez LA, Montero D. Association between selective serotonin reuptake inhibitors and upper gastrointestinal bleeding: population based case-control study. *BMJ.* 1999;319(7217):1106-1109.

3. Arnason T, Wells PS, van Walraven C, Forster AJ. Accuracy of coding for possible warfarin complications in hospital discharge abstracts. *Thrombosis research.* 2006;118(2):253-262.

4. Wahl PM, Rodgers K, Schneeweiss S, et al. Validation of claims-based diagnostic and procedure codes for cardiovascular and gastrointestinal serious adverse events in a commercially-insured population. *Pharmacoepidemiology and drug safety.* 2010;19(6):596-603.

5. Cunningham A, Stein CM, Chung CP, Daugherty JR, Smalley WE, Ray WA. An automated database case definition for serious bleeding related to oral anticoagulant use. *Pharmacoepidemiology and drug safety.* 2011;20(6):560-566.

6. Crooks CJ, Card TR, West J. Defining upper gastrointestinal bleeding from linked primary and secondary care data and the effect on occurrence and 28 day mortality. *BMC health services research.* 2012;12(1):392.

7. Valkhoff VE, Coloma PM, Masclee GM, et al. Validation study in four health-care databases: upper gastrointestinal bleeding misclassification affects precision but not magnitude of drug-related upper gastrointestinal bleeding risk. *Journal of clinical epidemiology.* 2014;67(8):921-931.

8. Friberg L, Skeppholm M. Usefulness of Health Registers for detection of bleeding events in outcome studies. *Thrombosis and haemostasis.* 2016;116(6):1131-1139.

9. Pasea L, Chung SC, Pujades-Rodriguez M, et al. Personalising the decision for prolonged dual antiplatelet therapy: development, validation and potential impact of prognostic models for cardiovascular events and bleeding in myocardial infarction survivors. *Eur Heart J.* 2017;38(14):1048-1055.

10. Mehran R, Rao SV, Bhatt DL, et al. Standardized Bleeding Definitions for Cardiovascular Clinical Trials: A Consensus Report From the Bleeding Academic Research Consortium. *Circulation.* 2011;123(23):2736-2747.

11. Schulman S, Kearon C. Definition of major bleeding in clinical investigations of antihemostatic medicinal products in non-surgical patients. *Journal of thrombosis and haemostasis : JTH.* 2005;3(4):692-694.

12. Wiviott SD, Antman EM, Gibson CM, et al. Evaluation of prasugrel compared with clopidogrel in patients with acute coronary syndromes: design and rationale for the TRial to assess Improvement in Therapeutic Outcomes by optimizing platelet InhibitioN with prasugrel Thrombolysis In Myocardial Infarction 38 (TRITON-TIMI 38). *American heart journal.* 2006;152(4):627-635.
